# Does Weaver–Dunn procedure have a role in chronic acromioclavicular dislocations? A meta-analysis

**DOI:** 10.1186/s13018-022-02995-9

**Published:** 2022-02-15

**Authors:** Hao-Ming Chang, Chi-Hsiu Wang, Kai-Lan Hsu, Fa-Chuan Kuan, Yueh Chen, Wei-Ren Su, Chih-Kai Hong

**Affiliations:** 1 Department of Orthopaedics, Tainan Municipal Hospital (Managed By Show Chwan Medical Care Corporation), Tainan, Taiwan; 2 Department of Nursing, National Cheng Kung University Hospital, College of Medicine, National Cheng Kung University, Tainan, Taiwan; 3 Department of Orthopaedic Surgery, National Cheng Kung University Hospital, College of Medicine, National Cheng Kung University, No. 138, Sheng-Li Road, Tainan City, 70428 Taiwan; 4 Department of Biomedical Engineering, National Cheng Kung University, Tainan, Taiwan; 5 Department of Orthopaedic Surgery, Sin Lau Hospital, Tainan, Taiwan; 6 Skeleton Materials and Bio-Compatibility Core Lab, Research Center of Clinical Medicine, National Cheng Kung University Hospital, College of Medicine, National Cheng Kung University, Tainan, Taiwan; 7 Musculoskeletal Research Center, Innovation Headquarter, National Cheng Kung University, Tainan, Taiwan

**Keywords:** Weaver–Dunn, Acromioclavicular joint, Reconstruction

## Abstract

**Background:**

In treatment of chronic acromioclavicular (AC) joint dislocations, both the Weaver–Dunn procedure (WD) and CC ligament reconstruction (CCR) are recommended options due to the low possibility of healing of the coracoclavicular (CC) ligaments. The aim of this review was to determine whether CCR will yield favorable clinical and radiographic outcomes in the treatment of chronic AC dislocations.

**Method:**

The Cochrane Library, EMBASE, and PubMed databases were searched for literature on chronic AC dislocations from data inception to June 30, 2021. Patient data were pooled using standard meta-analytic approaches. The Cochrane-Mantel–Haenszel method and variance-weighted means were used to analyze the outcomes. The Review Manager version 5.3 software (The Nordic Cochrane Centre, The Cochrane Collaboration, Copenhagen, Denmark) was used to calculate the heterogenicity, mean difference, and relative risk (RR) for all outcomes in the meta-analysis.

**Results:**

The current analysis included four trials on this topic, and all AC joint dislocations were classified as Rockwood types III to VI. The pooled data showed that the CCR group had significantly better post-operative American Shoulder and Elbow Surgeons Shoulder (ASES) scores, Oxford Shoulder Scores (OSSs), and Nottingham Clavicle Scores (NCSs) than the WD group, with a significant difference (*p* < 0.001, *p* = 0.020, and *p* < 0.001, respectively). In terms of the post-operative Constant-Murley Scores (CMSs), there were no significant differences between the CCR group and the WD group (*p* = 0.100). The CCR group had significantly better post-operative abduction and flexion of the index shoulder than the WD group (*p* < 0.001 and *p* < 0.001, respectively). In terms of radiological outcomes, the post-operative coracoclavicular distance (CCD) with a 10 kg load was smaller in the CCR group compared to that in the WD group (*p* < 0.001). The overall surgical wound infection rate was 11.6% in the WD group and 12.9% in the CCR, respectively (*p* = 0.82).

**Conclusion:**

The CCR group had better clinical outcome scores in the ASES, OOS, NCS, abduction, flexion, and external rotation than the WD group. In terms of radiological outcomes, the CCR group showed less displacement in weight-loaded post-CCD than the WD group, which indicated that the CCR provided more stability and resistance to deformation forces.

## Introduction

There are numerous variations in surgical techniques when treating symptomatic chronic acromioclavicular (AC) joint dislocations after failure of conservative treatment, each with their own respective advantages and disadvantages. The surgical techniques include AC and coracoclavicular reconstruction (CCR), with either biological or artificial grafts [1–4]), ligament transfers, such as coracoaromial ligament transfers, the Weaver–Dunn (WD) procedure [2, 5, 6], conjoined tendon transfers [7]), and various fixation techniques [8–12].

The Weaver–Dunn technique and CC ligament reconstruction are recommended options for treatment of chronic AC dislocations due to the low possibility of healing of the CC ligaments in chronic AC dislocations [13]. The WD procedure with several modifications including combined augmentation techniques is popular and widely accepted, as reported by previous studies due to favorable outcomes and low-to-moderate complications for treating chronic AC joint dislocations [2, 14, 15]. In addition, CC ligament reconstruction restores anatomical structure, and based on biomechanical studies, can provide more AC joint stability [16–18] and may lead to better clinical outcomes [19–22]. Recently, some studies compared the clinical and radiographic outcomes between the Weaver–Dunn procedure and CC ligament reconstruction in treatment of chronic AC dislocations, for which the findings indicated superior outcomes with CC ligament reconstruction [2, 5, 6, 23]. However, various assessments of clinical and radiological outcome measures were used in aforementioned studies, which lead to a lack of integration.

Therefore, the purpose of this meta-analysis was to comprehensively assess and compare clinical and radiographic outcomes for the WD procedure and CC ligament reconstruction in treatment of chronic AC dislocations. It is hypothesized that CC ligament reconstruction will yield favorable clinical and radiographic outcomes in the treatment of chronic AC dislocation.

## Method

### Search strategy

**Fig. 1:**
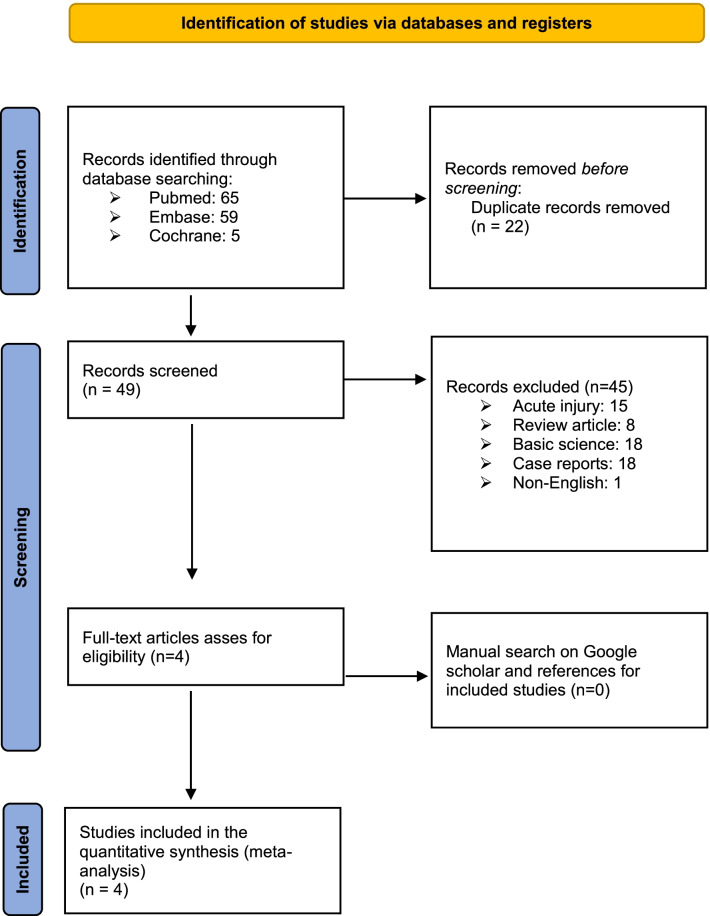
Flow diagram for study selection following the PRISMA (Preferred Reporting Items for Systematic Reviews and Meta-Analysis) guidelines

Three online databases (PUBMED, EMBASE, and Cochane) were searched for literature on chronic AC dislocations from data inception to June 30, 2021. The search terms included “acromioclavicular joint,” “reconstruction,” “Weaver–Dunn,” and similar phrases (Fig. 1). After the database search, the keywords were then entered into the Google Scholar website to determine if some articles had been missed. The inclusion criteria included (1) chronic AC dislocation using either the WD procedure or CC reconstruction, (2) human studies, (3) English language. The exclusion criteria included (1) acute AC injury (< 4 weeks), (2) review article, (3) non-surgical treatment, (4) fixation treatment, (5) cadaver/non-human studies.

### Study screening

A systematic screening approach based on the preferred Reporting Items for Systematic Reviews and Meta-analyses (PRISMA) [24] from title to full-text screening stages in duplicate was used by two independent reviewers (H-M C and C-K H). The third reviewer (F-C K) evaluated the possible discrepancies and decided if the studies should be included after comprehensive discussion. The references of the included studies were re-screened again using the same systematic approach to prevent missing any additional associated articles.

### Quality assessment

Two authors (H-M C and C-K H) evaluated the study quality and risk of bias using the STrengthening the Reporting of OBservational studies in Epidemiology (STROBE) guidelines for rating included studies [25]. The STROBE rating score of the included studies is shown in Table 1. The studies achieved high STROBE rating scores, thus indicating a low risk of bias.

**Table 1:** Overview of included studies in the meta-analyses

^Study^	^Study design, level of evidence^	^Mean injury to surgery (Mo)^	^Follow-up duration (Mo)^	^Subgroup^	^Patients, N^	^Mean age, Y^	^Gender^ ^M/F^	^Fracture type^	^Complication^	^STROBE^
^Tauber, 2009^	^Prospective cohort study, II^	^24 (6–144)^	^37 (24–58)^	^WD^	^12^	^42.6 ±10.1^	^8/4^	^Type III: 7^ ^Type IV: 2^ ^Type V: 3^	^Superficial SWI: 1^	^21/22^
				^CCR with autogenous STG^	^12^	^41.6 ±10.5^	^6/6^	^Type III: 5^ ^Type IV: 3^ ^Type V: 4^	^Superficial SWI: 0^	
^Kumar, 2014^	^Retrospective cohort study, III^	^39^	^40^	^WD^	^31^	^42 (19–72)*^	^N/A^	^Type III: 38^ ^Type IV: 8^ ^Type V: 9^	^Superficial SWI: 3^ ^LOR: 3^	^18/22^
				^CCR with Surgilig synthetic ligament^	^24^		^N/A^	^N/A^	^Superficial SWI: 4^ ^LOR: 1^	
^Hegazy, 2016^	^Prospective cohort study, II^	^18.2(9–28)^	^27.8 (24–32)^	^WD^	^10^	^40.3±13.6^	^9/1^	^Type III: 10^	^Superficial SWI: 3^ ^LOR: 3^	^17/22^
				^CCR with autogenous STG^	^10^	^37.9±9.6^	^8/2^	^Type III: 10^	^Superficial SWI: 4^ ^LOR: 0^	
^Kocaoglu, 2017^	^Retrospective cohort study, III^	^N/A^	^44.9 (29–60)^	^WD with dynamic TightRope system^	^16^	^37.9±10.5^	^14/2^	^Type III:13^ ^Type IV: 2^ ^Type V: 1^	^Superficial SWI: 1^	^19/22^
				^CCR with autologous PLG^	^16^	^41.4±8.3^	^13/3^	^Type III: 12^ ^Type IV: 2^ ^Type V: 1^ ^Type VI: 1^	^Superficial SWI: 0^	

WD, Open modified Weaver–Dunn procedure; CCR, coracoclavicular reconstruction; STG, semitendinosus tendon graft; PLG, peroneal longus graft; SWI, surgical wound infection; LOR, Loss of reduction

*Mean age with range in all patients, subgroups data not provide

### Data abstraction

Demographic data are shown in Table 1 and include the author, year, publication, sample size, study design, and patient demographics. We evaluated all preoperative and postoperative outcomes (including clinical and radiographic outcomes), and complications were also documented.

### Statistical analysis

We used the Cochrane-Mantel–Haenszel method and variance-weighted means to analyze the outcomes. The effects of heterogeneity were evaluated using the *I*^2^ value (ranging from 0 to 100%), where *I*^2^ > 50% indicated obvious heterogeneity [26, 27]. If studies showed heterogeneity, a random-effects analysis was used to compare groups [28]. Otherwise, a fixed-effects analysis was used for comparing studies without obvious heterogeneity [29]. Review Manager version 5.3 software (The Nordic Cochrane Centre, The Cochrane Collaboration, Copenhagen, Denmark) was used to calculate the heterogenicity, mean difference, and relative risk (RR) of all outcomes in the meta-analysis.

## Results

### Study characteristics

Initially, a total of 65 articles was found using the search strategy discussed above. After excluding 22 duplicates, 49 studies were included. After applying the inclusion and exclusion criteria, a systemic screening process enrolled four studies that met the inclusion criteria, which included two prospective cohort studies (Level II) and two retrospective cohort studies (Level III) (Fig. 1).

### Patient characteristics

A total of 69 patients receiving the WD procedure and 62 patients receiving CCR were included in the meta-analysis. The characteristics of the patients in the four studies are summarized in Table 1. Among the studies, all AC joint dislocations were classified as Rockwood types III to VI. In the Weaver–Dunn procedure, all included studies used an open modified method [2, 5, 6, 23]. In the CCR group, in two of the studies, autogenous semitendinosus grafts (STG) were used [2, 5]; one used an allogenous peroneal longus graft (PLG), and another one used a synthetic ligament [6].

### Meta-analysis

**Fig. 2:**
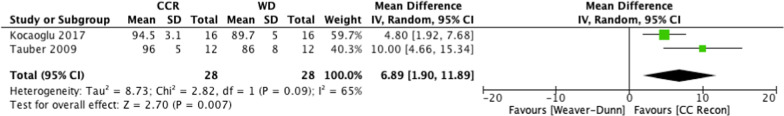
Comparison of the mean postoperative American Shoulder and Elbow Surgeons Shoulder (ASES) scores for the Weaver–Dunn procedure (WD) and coracoclavicular reconstruction (CCR) in chronic acromioclavicular dislocations. (95% CI confidence interval)

**Fig. 3:**
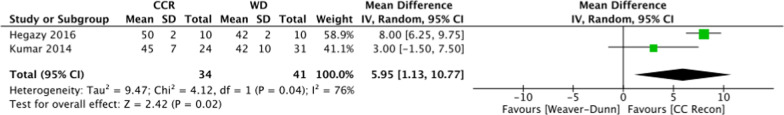
Comparison of the mean postoperative Oxford Shoulder Scores for the Weaver–Dunn procedure (WD) and coracoclavicular reconstruction (CCR) in chronic acromioclavicular dislocations. (95% CI confidence interval)

**Fig. 4:**
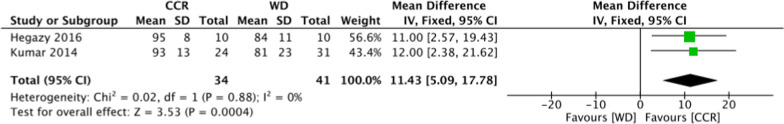
Comparison of the mean postoperative Nottingham Clavicle Scores for the Weaver–Dunn procedure (WD) and coracoclavicular reconstruction (CCR) in chronic acromioclavicular dislocations. (95% CI confidence interval)

**Fig. 5:**
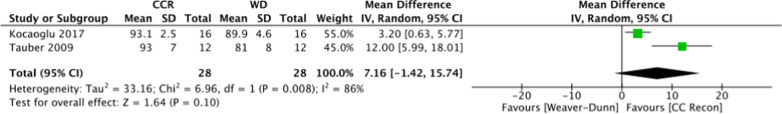
Comparison of the mean postoperative Constant-Murley Score for the Weaver–Dunn procedure (WD) and coracoclavicular reconstruction (CCR) in chronic acromioclavicular dislocations. (95% CI confidence interval)

The pooled data showed that the CCR group had significantly better post-operative American Shoulder and Elbow Surgeons Shoulder Scores (ASESs), Oxford Shoulder Scores (OSSs), and Nottingham Clavicle Scores (NCSs) than the WD group, with a significant difference (post-ASES, 95.1 versus 87.9, 95% CI 1.90–11.89, *p* = 0.0007; post-OOS, 46.5 versus 42.0, 95% CI 1.13–10.77, *p* = 0.02; post-NCS, 93.6 versus 81.7, 95% CI 5.09–17.78, *p* = 0.0004) (Figs. 2, 3, 4). In terms of the post-operative Constant-Murley Scores (CMSs), the CCR group had a trend toward a better CMS compared to the WD in the pooled data, but it did not achieve a statistically significant difference (Fig. 5) (post-CMS, 92.9 versus 86.2, 95% CI − 1.42–15.74, *p* = 0.10).

**Fig. 6:**
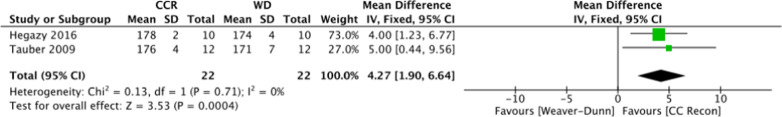
Comparison of the mean postoperative abduction for the Weaver–Dunn procedure (WD) and coracoclavicular reconstruction (CCR) in chronic acromioclavicular dislocations. (95% CI confidence interval)

**Fig. 7:**
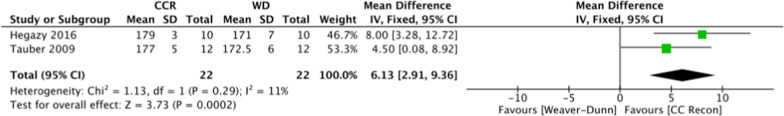
Comparison of the mean postoperative flexion for the Weaver–Dunn procedure (WD) and coracoclavicular reconstruction (CCR) in chronic acromioclavicular dislocations. (95% CI confidence interval)

**Fig. 8:**
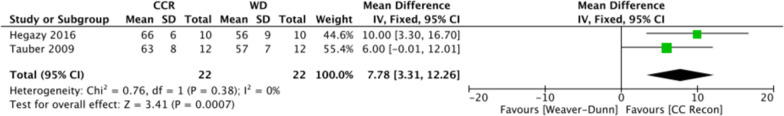
Comparison of the mean postoperative external rotation for the Weaver–Dunn procedure (WD) and coracoclavicular reconstruction (CCR) in chronic acromioclavicular dislocations. (95% CI confidence interval)

In terms of range of motion, the CCR group showed better post-operative abduction (post-ABD), flexion (post-FLEX), and external rotation (post-ER) of the index shoulder than the WD group with a statistical difference (post-ABD, 176.9 versus 172.4 degree, 95% CI 1.90–6.64, *p* = 0.0004; post-FLEX, 177.9 versus 171.8 degree, 95% CI 2.91–9.36, *p* = 0.0002; post-ER, 64.4 versus 56.5 degree, 95% CI 3.31–12.26, *p* = 0.0007) (Figs. 6, 7, 8).

**Fig. 9:**
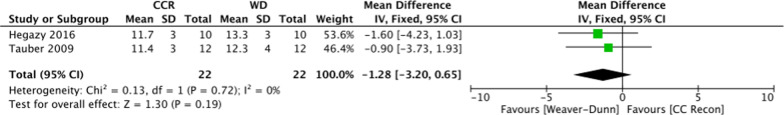
Comparison of the mean postoperative coracoclavicular distance for the Weaver–Dunn procedure (WD) and coracoclavicular reconstruction (CCR) in chronic acromioclavicular dislocations. (95% CI confidence interval)

**Fig. 10:**
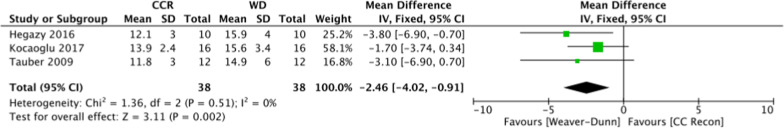
Comparison of the mean post-operative coracoclavicular distance (post-CCD) with 10 kg load for the Weaver–Dunn procedure (WD) and coracoclavicular reconstruction (CCR) in chronic acromioclavicular dislocations. (95% CI confidence interval)

In terms of the radiological outcomes, the mean of the post-operative CCD showed no statistical between-group differences (11.5 mm in CCR group versus 12.8 mm in WD group, 95% CI − 3.2–0.65, *p* = 0.19). However, in the post-operative CCD with a 10 kg load, the CCR group had less post-CCD distance than the WD group, with significant between-group differences (12.8 mm and 15.45 mm, respectively, 95% CI − 4.02 ~ − 0.91, *p* = 0.0002) (Figs. 9, 10).

**Fig. 11:**
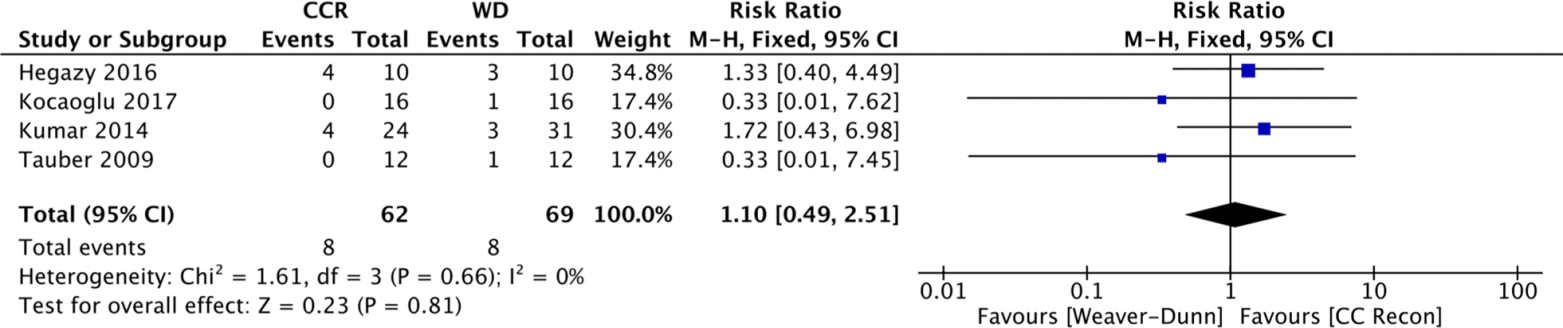
Comparison of the mean difference of post-operative wound infections for the Weaver–Dunn procedure (WD) and coracoclavicular reconstruction (CCR) in chronic acromioclavicular dislocation

No major complications were noted in the included studies. The overall surgical wound infection rate was 12.2%, with 11.6% in the WD group and 12.9% in the CCR group, respectively. The pooled data showed no significant between-group differences in the rate of surgical wound infection (RR 1.10; 95% CI 0.49–2.51, *p* = 0.81) (Fig. 11).

## Discussion

Both the WD procedure and the CCR procedure achieved satisfactory outcomes in terms of treating chronic AC dislocations [2, 23, 30, 31]. Some studies supported the use of the WD procedure [14, 15, 30–32] whereas others favored CCR techniques [2, 5, 6, 23]. A meta-analysis was further conducted to compare the outcomes for the WD procedure and CCR in the treatment of chronic AC dislocations. The results showed that CCR led to better functional outcomes and appeared to provide better stability in terms of maintaining reduction than the WD procedure.

The original WD procedure included a lateral clavicle resection, reduction of the dislocated clavicle, and transfer of the coracoacromial ligament to the lateral clavicle without additional fixation [33]. Later, numerous studies discussed modified WD procedures using various additional fixation devices intended to provide further stability and maintain reduction of the AC joint, in which satisfactory clinical and radiological outcomes were achieved [32, 34–36]. One study reported that 75% of the modified WD procedures had good to excellent results [36, 37]. However, the disadvantages of the WD procedure included the fact that the initial strength of the transferred CA ligament was 25% weaker than the normal CC ligament, and it was impossible to control horizontal stability, which suggests recurrent subluxation and dislocation [37, 38]. The results from the current study suggest that although there were between-group differences in the post-CCD distance for the WD and CCR groups, the WD group had a greater post-CCD distance than the CCR group with weight loading. This finding may indicate that the WD procedure led to recurrent subluxation and dislocation during follow-up, which concurred with previous findings.

In the last 10 years, treating chronic AC dislocations with the CCR procedure has become increasingly more popular [31, 39]. Many studies have introduced the use of autogenous, allogenic free tissue grafts, or synthetic grafts to reconstruct CC ligaments anatomically, which has become a trend in the treatment of chronic AC joint dislocations [1, 9, 23]. In previous biomechanical studies, anatomical CCR reconstruction was shown to more closely resemble the original stiffness of the native CC ligaments and to provide more AC joint stability than the WD procedure [40, 41]. However, the disadvantages of CCR include distal clavicle fractures, coracoid fractures, graft ruptures, and donor site morbidity if an autogenous grafts was selected [42, 43]. In the present study, the CCR group had better clinical outcome scores in the ASES, OOS, NCS, abduction, flexion, and external rotation, with the exception of the CMSs. In terms of radiological outcomes, post-CCD showed no between-group differences, but the CCR group showed less displacement in the weight-loaded post-CCD than the WD groups, which indicated that the CCR provided more stability and better resistance to deformation forces.

Common postoperative complications of the WD procedure included recurrent instability (especially in the anteroposterior direction), loss of reduction, augmented implant failures, surgical site infections, foreign body reactions, higher unplanned reoperation rates, and heterotopic calcification [31, 44–46]. In the CCR procedure, common postoperative complications included donor site morbidity for tendon autografts, clavicle or coracoid fractures, heterotopic calcification, AC joint arthritis/osteolysis, adhesive capsulitis, surgical site infections, loss of reduction, clavicular bone tunnel widening, and implant failures [2, 44, 47, 48]. In the present meta-analysis, the postoperative complications in the WD and CCR procedures were compared and analyzed, where it was found that the CCR group had less post-CCD distance than the WD group with statically significant between-group differences, which concurred with the findings of previous studies. Hence, surgeons should be aware of the potential for postoperative losses in reduction after performing a WD procedure.

## Limitations

There were several limitations in this meta-analysis. First, the sample sizes in the enrolled studies were relatively small. In addition, two of the enrolled studies were retrospective designs, which could have had some potential biases. Therefore, more large-scale, prospective randomized studies may be needed in the future to provide evidence in the treatment outcomes for chronic AC dislocation. Second, there is no homogenous consensus about the definition of a “chronic” AC dislocation in terms of the time from injury to surgery among the searched studies, where the duration ranged from three weeks to six months [1, 3, 5, 10, 49]. This heterogenicity may contribute to bias in these types of meta-analyses. However, a recent study showed a high degree of consensus suggesting that the separation line between acute and chronic cases could be set at three weeks, and all studies included in the present study met this criteria [39]. Third, in the CCR article, different types of ligament grafts were included among the articles reviewed in this study, which may have led to within-group differences. A recent systemic review revealed that allo- and autografts had comparable outcomes in chronic acromioclavicular joint reconstruction [50], and another study revealed comparable functional results between anatomical synthetic and biologic reconstructions [51].

## Conclusion

In this meta-analysis, both the WD procedure and CCR achieved satisfactory results in treatment of chronic AC dislocations. The CCR group yielded better clinical outcome scores in the ASES, OOS, NCS, abduction, flexion, and external rotation than the WD group. In terms of radiological outcomes, the CCR group showed less displacement in weight-loaded post-CCD than the WD group, which indicated that the CCR provides more stability and resistance to deformation force.

## Data Availability

The datasets used and analyzed during the current study are available from the public online database.

## Abbreviations

ACJ: Acromioclavicular joint
ASES: American shoulder and elbow surgeons shoulder scores
CC: Coracoclavicular
CCD: Coracoclavicular distance
CCR: Coracoclavicular ligament reconstruction
CMSs: Constant-Murley scores
CI: Confidence interval
NCSs: Nottingham Clavicle scores
OSSs: Oxford Shoulder Scores
PRISMA: Preferred reporting items for systematic reviews and meta- analyses
RR: Relative risk
STROBE: Strengthening the reporting of observational studies in epidemiology
WD: Weaver–Dunn procedure
